# Assessment of biomarkers of thyroid hormone action across three thyroid hormone replacement regimens in hypothyroidism

**DOI:** 10.3389/fendo.2026.1919606

**Published:** 2026-08-03

**Authors:** Betty Ann Bjerkreim, Sara Salehi Hammerstad, Sindre Lee-Ødegård, Erik Fink Eriksen

**Affiliations:** 1 Department of Medical Biochemistry, Oslo University Hospital, Oslo, Norway; 2 Department of Endocrinology, Morbid Obesity and Preventive Medicine, Oslo University Hospital, Oslo, Norway; 3 Institute of Clinical Medicine, Faculty of Medicine, University of Oslo, Oslo, Norway; 4 Pilestredet Park Specialist Center, Oslo, Norway

**Keywords:** biomarkers, combination therapy, hypothyroidism, safety markers, T3 monotherapy, thyroid hormone efficacy

## Abstract

**Background:**

A common concern during thyroid hormone replacement therapy is over-substitution leading to increased risk of primarily osteoporotic fracture, and adverse cardiac outcomes in particular. The addition of synthetic triiodothyronine (T3) to treatment regimens for hypothyroidism has been singled out as being associated with a higher risk of such adverse effects. While thyroid-stimulating hormone (TSH) has been a universally accepted, useful parameter for monitoring treatment responses in thyroid diseases, it is not perfect. This is highlighted by the fact, that 5-10% of patients with hypothyroidism continue to exhibit hypothyroid symptoms, despite normal circulating TSH levels.

**Methods:**

While assessment of intracellular T3 concentrations is impossible, biomarkers reflecting the cellular action of intracellular T3 binding to nuclear receptors may be informative than assessing extracellular thyroid hormone concentrations. The aim of this study was therefore twofold: 1) to investigate the variation between TSH levels and four biomarkers related to thyroid hormone action (N-terminal pro b-type natriuretic peptide (NT-proBNP), N-terminal propeptide of type I collagen (PINP), sex hormone binding globulin (SHBG) and low-density lipoprotein cholesterol (LDL-C) with different TSH levels and 2) to assess whether these biomarkers differed among patients receiving three different treatment regimens: synthetic thyroxine (T4) monotherapy, T3 monotherapy and T4/T3 combination therapy.

**Results:**

SHBG revealed a steady significant increase within the reference range with decreasing TSH (p<0.001), with the highest values being reached at TSH < 0.01 mIU/L. LDL-C, however, did not show significant variation with TSH. NT-proBNP also revealed a significant increase within the reference range with decreasing TSH (p= 0.002), while PINP exhibited an inverse relationship (p=0.042). When analyzing biomarkers in relation to treatment regimen, the following patterns emerged: In patients on T3 monotherapy, SHBG was significantly higher (p<0.001), and LDL-C significantly lower (p=0.045) compared to the other groups. NT-proBNP and PINP, however, did not show any significant differences between the three treatment groups.

**Discussion:**

Our results suggest that in a subgroup of hypothyroid patients, with persistent hypothyroid symptoms, T3 monotherapy may further enhance the clinical effects of thyroid hormone substitution therapy, without increasing the risk of adverse effects at the heart and skeleton.

## Introduction

The main concern during thyroid hormone replacement therapy is over-substitution leading to increased risk of osteoporotic fracture (1–3), and adverse cardiac outcomes, in particular atrial fibrillation and heart failure (4). The addition of synthetic triiodothyronine (T3) to treatment regimens in hypothyroidism has been singled out as being associated with a higher risk of such side effects, mainly due to concerns pertaining to varying and excessive T3 levels during therapy (5).

While thyroid-stimulating hormone (TSH) has been a universally accepted, useful parameter reflecting treatment responses in thyroid diseases, it is not perfect. It reflects feedback from circulating thyroxine (T4) and T3 on the pituitary and hypothalamus, but not necessarily other organs with potentially different set-points for cellular effects. These limitations of TSH as a response parameter is highlighted by the fact that 5-10% of patients with hypothyroidism continue to exhibit hypothyroid symptoms, especially cold intolerance, fatigue, cognitive problems and myalgias, despite normal circulating TSH, T4 and T3 levels (5–7).

As highlighted in the rodent study by Escobar-Morreale et al., tissue levels of T3 and T4 vary considerably between different organs, and normal circulating T4 and T3 levels do not secure sufficient intracellular T3 levels in some tissues, with particular discrepancies between T4 and T3 concentrations in the cerebral cortex and brown adipose tissue (8). It is therefore conceivable that the sensitivity to, and effects of thyroid hormones in other organs like the brain, heart and skeleton may be different from that of the pituitary. Additional use of other biomarkers reflecting post-receptor effects of thyroid hormone at the cellular level might therefore be more informative than relying on TSH alone. Moreover, such assessment could potentially lead to more personalized treatment. We and others have previously found that low-density lipoprotein cholesterol (LDL-C) and sex hormone binding globulin (SHBG) are dynamic efficacy parameters reflecting cellular effects of T3 binding to intracellular receptors (9, 10).

N-terminal pro b-type natriuretic peptide (NT-proBNP) has been shown to be a sensitive marker of diverse adverse cardiac effects, including those elicited by thyroid hormone excess like tachycardia, atrial fibrillation and heart failure (11–13). In the skeleton, thyroid hormone excess causes increased bone turnover with a negative remodeling balance (14). This leads to accelerated bone loss and increased risk of osteoporotic fracture (3, 15, 16). In this context, the bone turnover marker N-terminal Propeptide of type I Procollagen (PINP) has emerged as a very sensitive marker of bone remodeling activity in metabolic bone disease (17), including hypo- and hyperthyroidism (18). Contrary to other bone turnover markers, it is unaffected by food intake and shows insignificant diurnal variation (17).

The aim of this study was twofold: (1) to investigate the variation of these biomarkers (PINP, NT-proBNP, LDL-C, and SHBG) across TSH levels; and (2) to assess whether biomarkers primarily reflecting T3 efficacy (LDL-C and SHBG; hereafter referred to as “efficacy parameters”) and biomarkers primarily reflecting organ-level adverse effects (PINP and NT-proBNP; hereafter referred to as “safety parameters”) differed among patients receiving three thyroid hormone replacement regimens: T4 monotherapy, T3 monotherapy, and T4/T3 combination therapy.

## Materials and methods

### Study design and patients

In total, 132 patients (mean age 51 years (range 26-81) undergoing thyroid hormone substitution therapy for hypothyroidism were sampled consecutively in our clinic. Hypothyroidism was caused by either thyroiditis or ablation through operation or radioiodine. Patients on T3 monotherapy and T4/T3 combination therapy had all been shifted from T4 monotherapy due to persistent hypothyroid symptom despite TSH levels within the reference range. Patients with known diseases or treated with pharmaceuticals known to affect NT-proBNP (heart failure, atrial fibrillation, renal disease, angiotensin converting enzyme inhibitors, angiotensin II receptor blockers), and PINP (other metabolic bone diseases, bisphosphonate, denosumab, osteoanabolic agents) were excluded. The collection of clinical data was approved by the regional ethical committee (ID 218347) and the study was performed in accordance with the declaration of Helsinki.

Clinical parameters and biomarkers were collected at two visits: at baseline and at a follow-up visit conducted at least one year later. This article reports only the laboratory results obtained at the follow-up visit. Forty-five assessments were from patients treated with T4 monotherapy, 96 assessments from patients on T3 monotherapy, 25 assessments from patients treated with T4/T3 combination therapy. All parameters were classified according to five different TSH categories (TSH < 0.01 (fully suppressed); TSH 0.01-0.3 (below lower limit of reference); TSH 0.3-2 (lower half of reference range); TSH 2-4 (upper half of reference range); and TSH >4 (above higher limit of reference) and in separate analyses according to treatment regimen.

### Laboratory analyses

Thyroid hormone tests: TSH (reference range: 0.2-4.0 mIU/L) was measured with a non-competitive immunofluorometric analysis by Autodelfia (Wallac Oy, Turku, Finland). Free T4 (8.0-21.0 pmol/L) was measured with solid-phase time-delayed fluoro-immunoassay with back-titration by Autodelfia (Wallac Oy, Turku, Finland). Free T3 (2.8-7.0 pmol/L) was measured with competitive electrochemiluminescence immunoassay by Cobas e601 (Roche Diagnostics, Indianapolis, IN, USA).

LDL-C (1.4-5.3 mmol/L) was measured by direct enzymatic colorimetric assay on the ADVIA Chemistry XPT system (Siemens Healthineers, Germany). SHBG (25–150 nmol/L) was measured using a sandwich immunoassay on the ADVIA Centaur XPT system (Siemens Healthineers, Germany), employing direct chemiluminescent technology. PINP (11-125 µg/L) was measured with a non-competitive electro-chemiluminescence immunoassay by Cobas e601 (Roche Diagnostics, Indianapolis, IN, USA). NT-proBNP (0–300 ng/L) was measured using a sandwich immunoassay on the ADVIA Centaur XPT system (Siemens Healthineers, Germany), employing direct chemiluminescent technology.

All analyses were performed at Fürst Medical Laboratory, Oslo.

### Statistical analyses

All statistical analyses were performed using IBM SPSS Statistics for Windows (Version 29; IBM Corp., Armonk, N.Y., USA). Statistical significance was defined as two-tailed *P* < 0.05. The differences between categorical TSH values and treatment groups on the continuous laboratory variables were analyzed by the Kruskal-Wallis test and the Mann-Whitney U test. All variables were tested for normality by quantile-quantile (QQ) plots and histogram.

## Results

**Table 1** presents the characteristics of patients receiving the three thyroid hormone replacement regimens. Patients on T4 monotherapy, T3 monotherapy and T4/T3 combination therapy exhibited TSH values within the normal range. As expected, the T3 monotherapy group revealed non-detectable free T4 levels, while free T3 levels were above the upper limit of normal. This is in accordance with the treatment goals employed in this group, which aims at achieving normal TSH and T3 levels in the range 6–10 nmol/l (6, 9). T4 levels would be expected to be undetectable in patients without thyroid gland function (6).

**Table 1:** Demographics of the populations studied.

	Reference range	T4 monotherapy	T3 monotherapy	T4/T3 combination therapy	P-value
N		45	115	25	
TSH mIU/L	0.2-4.0	0.65 (1.39)	0.33 (0.92)	0.29 (0.97)	0.030
FT4 pmol/L	8-24	17.0 (3.6)	0.0 (2.7)	15.0 (6.0)	<0.001
FT3 pmol/L	2,8-7,0	4.9 (0.8)	7.7 (2.2)	5.0 (2.4)	<0.001
SHBG nmol/L	20-150	55.5 (42.0)	83.5 (71.0)	78.0 (63.0)	0.003
LDL-C mmol/L	1,4-5,3	3.3 (1.3)	2.9 (1.3)	2.8 (1.6)	0.090
NT-proBNP ng/L	0-300	59.0 (48.0)	66.0 (83.0)	70.0 (55.0)	0.186
PINP µg/L	25-120	52.0 (21.0)	58.0 (30.0)	62.0 (43.0)	0.105

Data are presented as median (interquartile range).

T4, synthetic thyroxine; T3, lsynthetic triiodothyronine; TSH, thyroid stimulating hormone; FT4, free thyroxine; FT3, free triiodothyronine; SHBG, sex hormone binding globulin; LDL-C, low-density lipoprotein-cholesterol; NT-proBNP, N-terminal pro b-type natriuretic peptide; PINP, N-terminal Propeptide of type I Procollagen.

**Figure 1:**
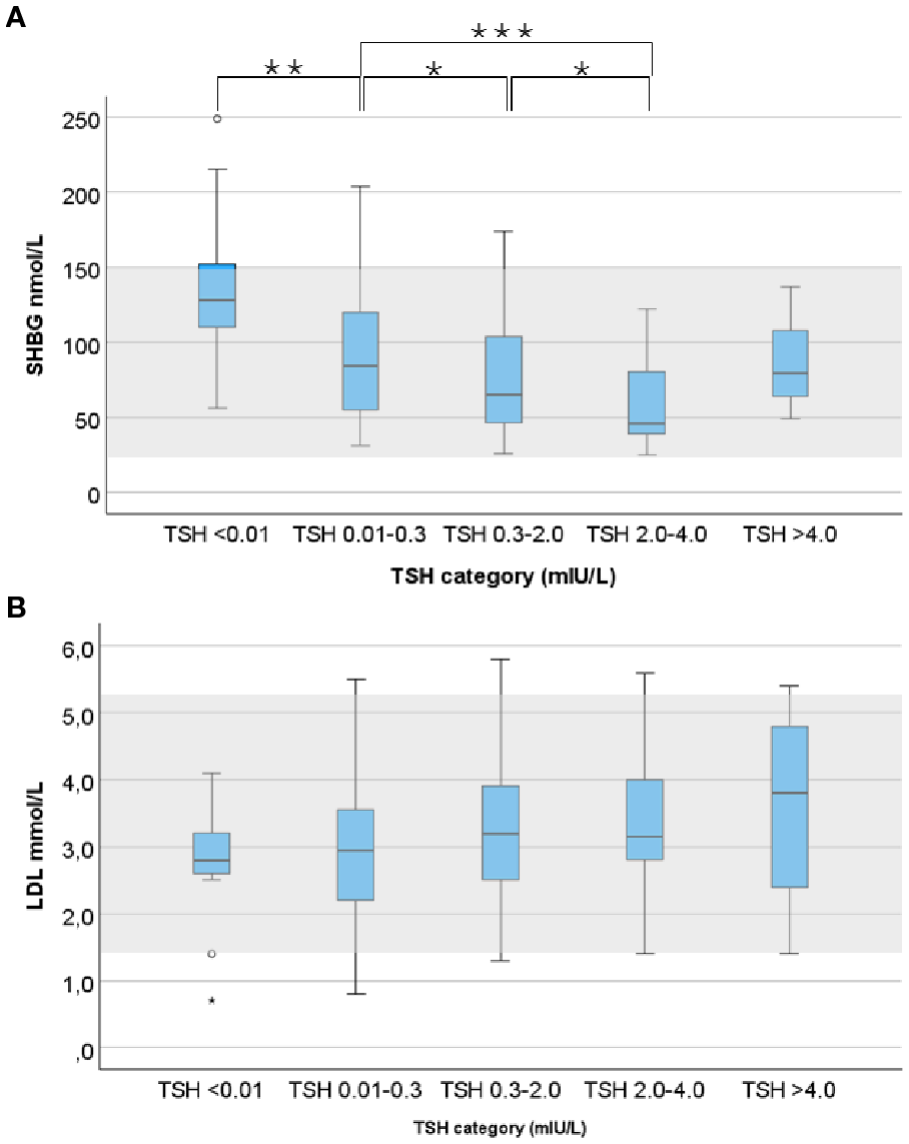
Variation in LDL-C **(a)** and SHBG **(b)** with TSH category. *p<0.05; **p<0.01; ***p<0.001.

**Figure 2:**
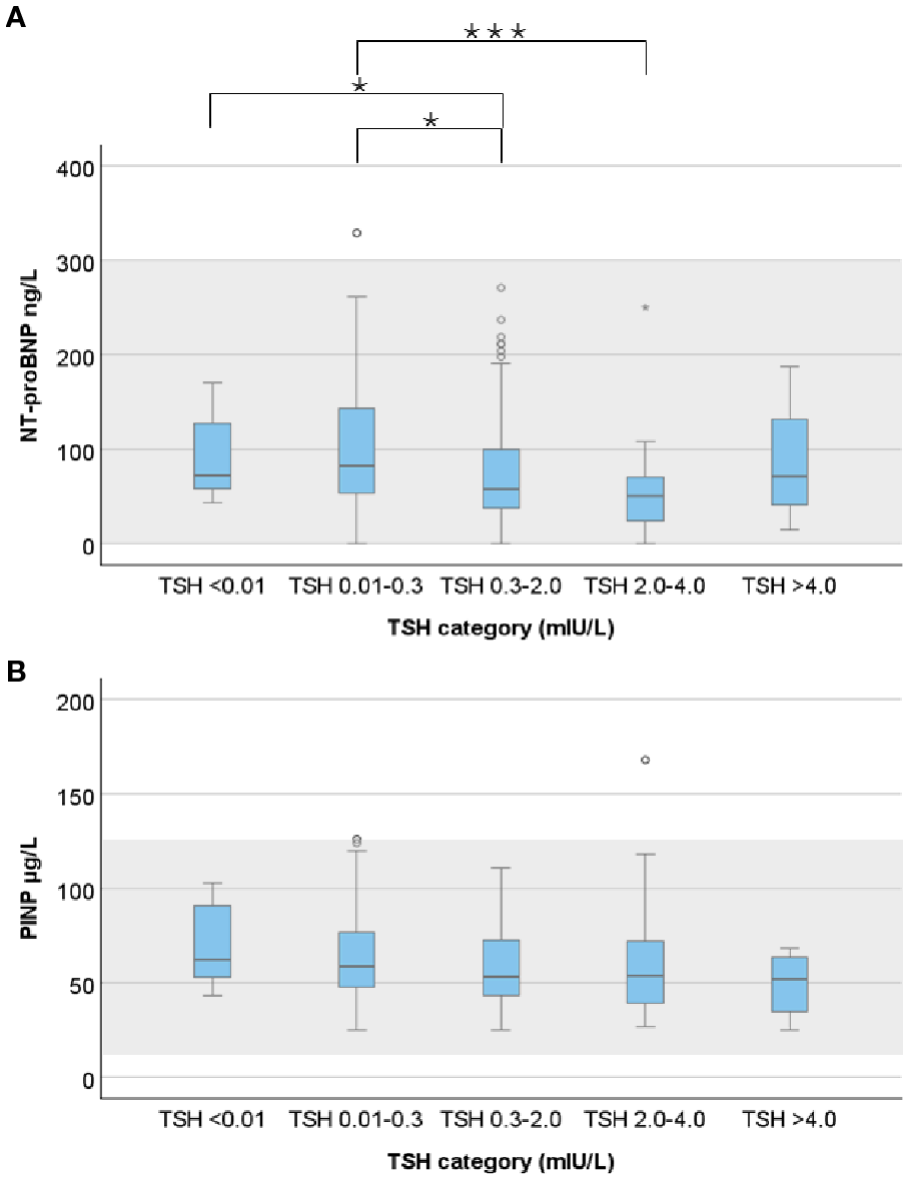
Variation in NT-proBNP **(a)** and PINP **(b)** with TSH category.

When analyzing efficacy and safety parameters according to TSH levels, the following differences emerged. SHBG revealed a steady significant increase with decreasing TSH within the reference range (p<0.001), with the highest values being reached at TSH < 0.01 mIU/L (**Figure 1a**). LDL-C, however, did not show significant variation with TSH (p=0.498) (**Figure 1b**). NT-proBNP also revealed a significant increase with decreasing TSH (p= 0.002) (**Figure 2a**), and one patient showed values above the upper limit. PINP exhibited the same inverse relationship to TSH (p=0.042). Six patients, all postmenopausal (age 50-82), exhibited values above the reference range (**Figure 2b**).

**Figure 3:**
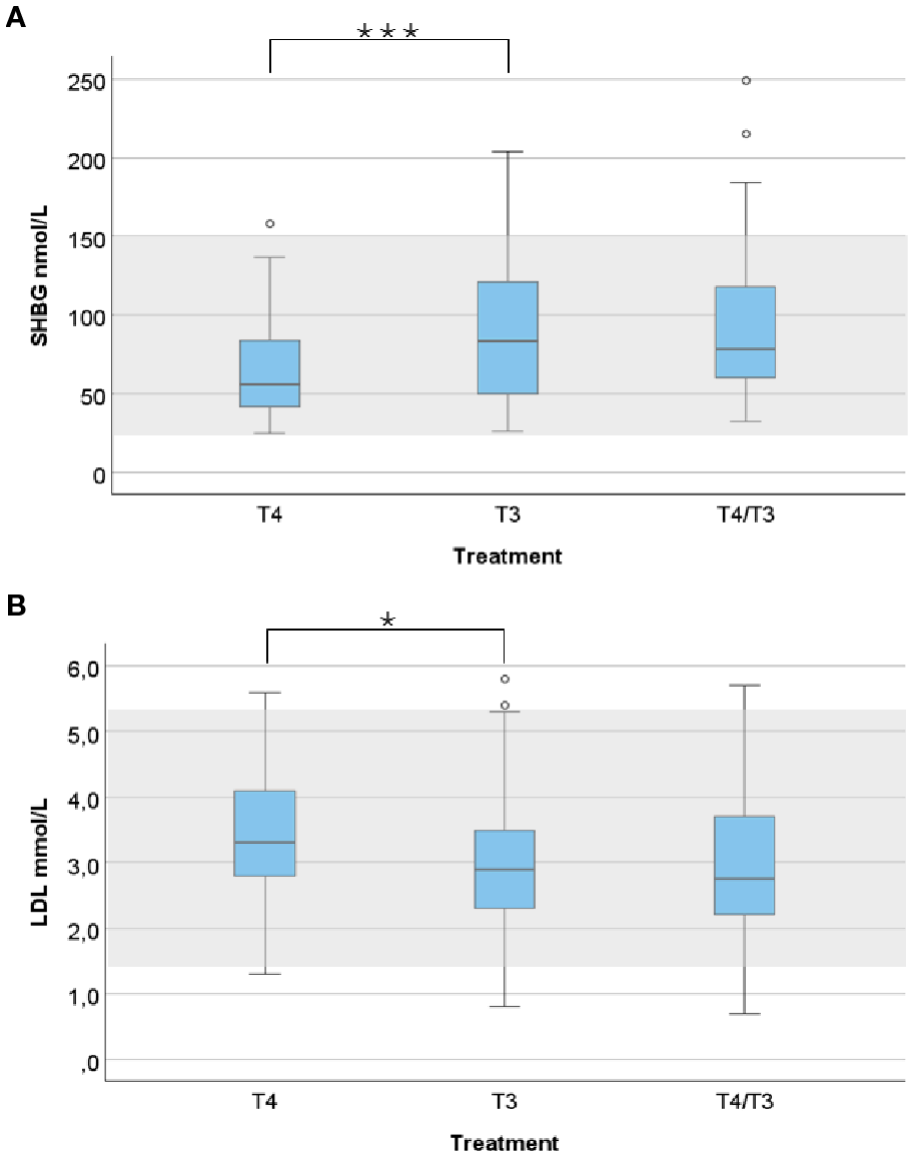
Variation of the efficacy biomarkers, SHBG **(a)** and LDL-C **(b)**, in relation to whether the patient was treated with T4 monotherapy, T3 monotherapy or T4/T3 combination therapy. *p<0.05; **p<0.01; ***p<0.001.

**Figure 4:**
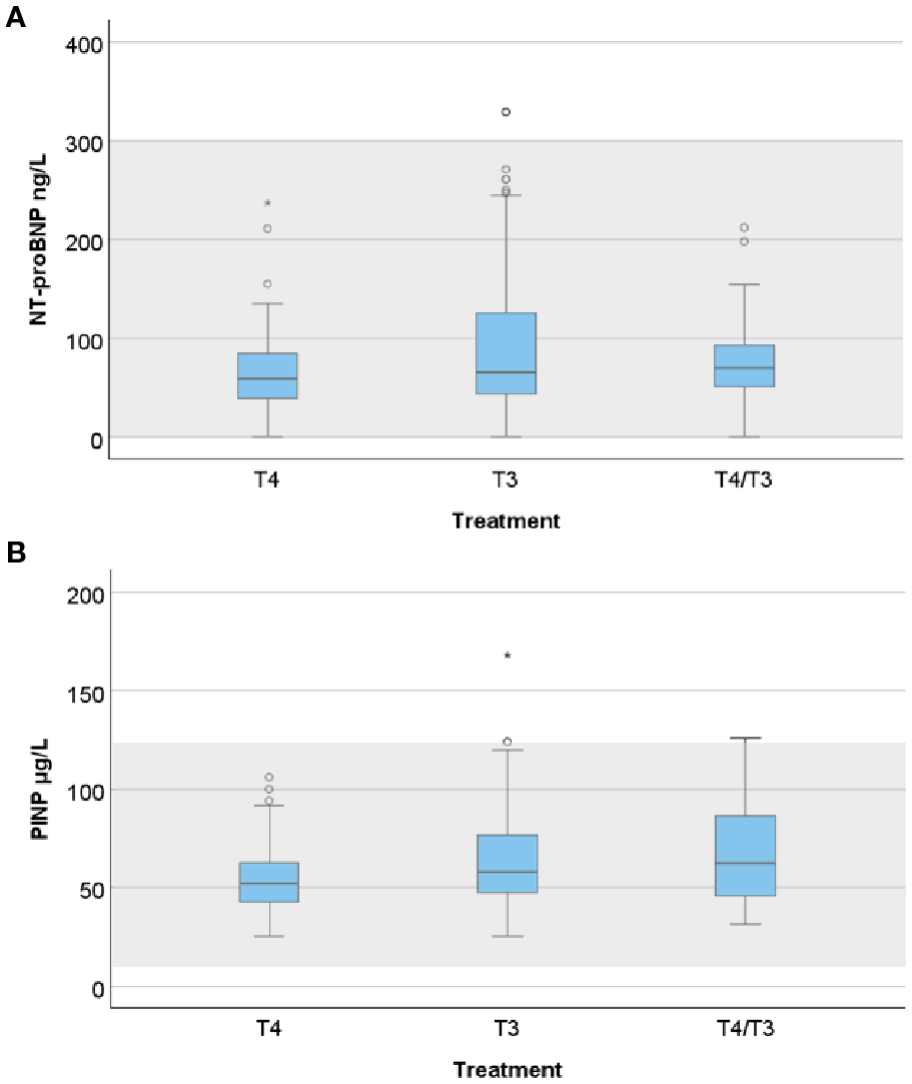
The variation of the safety biomarkers, NT-proBNP **(a)** and PINP **(b)**, in relation to whether the patient was treated with T4 monotherapy, T3 monotherapy or T4/T3 combination therapy. *p<0.05; **p<0.01; ***p<0.001.

When analyzing biomarkers in relation to treatment regimen the following patterns emerged: In patients on T3 monotherapy, SHBG (**Figure 3a**) was significantly higher (p<0.001), and LDL-C significantly lower (p=0.045) compared to the other groups. (**Figure 3b**). No other differences pertaining to SHBG or LDL-C were found between groups. The safety parameters NT-proBNP (**Figure 4a**) and PINP (**Figure 4b**), however, did not show any differences between the three treatment groups.

## Discussion

Treatment with T3, in combination with T4 or as T3 monotherapy, is generally considered to be associated with increased risk of cardiac and skeletal adverse effects, even though most analyses have failed to demonstrate significant safety issues. In studies employing a wide range of T4:T3 ratios on a variety of patient populations, with spontaneously occurring hypothyroidism or following thyroid ablation of thyroid tissue, only small and insignificant changes in heart rate or increased bone resorption were reported (19). One small study randomized 14 individuals with hypothyroidism to monotherapy with T4 or T3 for six weeks to achieve TSH in the low-normal range (0.5–1.5 mIU/L) and found no adverse changes in blood pressure or heart rate between the two groups (20). This is similar to our own data on cardiac effects in a larger group of patients on T3 monotherapy (6). A large retrospective observational study followed 400 individuals who had ever taken a T3 preparation and compared them to 33,955 individuals who had only taken T4 (average duration 17 years) (21). No increased risk of cardiac or skeletal adverse effects were seen, but T3 use was associated with an approximately twofold increase in prescriptions of antipsychotic drugs (21). The latter observation has not been reported in other studies.

A recent study looked at spontaneous adverse events reports arising for a T4/T3 combination therapy in eight countries (Austria, Belgium, Chile, France, Germany, Mexico, New Caledonia, and Poland) (19). The numbers of adverse events were very low, as the study found that the number of units sold to generate a single adverse event possibly relevant to thyrotoxicosis ranged from about 2.8 million to 178 million (19). In this context, it is worth noting that one case of supraventricular arrhythmias, a dominant symptom of thyrotoxicosis, was reported for each 39,314,895 units of the T4/T3 combination tablet sold (22).

In a study on post myocardial infarction patients T3 administration was associated with a nonsignificant increase in atrial fibrillation (23). Also, in a post-cardiac surgery population which has been associated with increased risk of atrial fibrillation due to a postoperative euthyroid sick syndrome, T3 supplementation actually reduced the risk of atrial fibrillation (24). Low T3 levels also seem to play a significant role in the pathogenesis of heart failure, but in such patients, T3 supplementation did not improve ejection fraction (25). The use of T3 was associated with increased incidence of heart failure and stroke in patients with a longer duration of T3 use and history of thyroid cancer (26). TSH levels were, however, generally suppressed, which may explain the discrepancy (26).

Past research has shown NT-proBNP to be a sensitive marker of both heart failure, supraventricular arrythmias, in particular atrial fibrillation (27). In the current study, NT-proBNP did not deviate significantly from the reference range in neither of the three treatment regimens studied as long TSH remained within the reference range.

In our hands, SHBG emerged as the most dynamic biochemical efficacy parameter reflecting intracellular T3 action, showing a significant increase with lower TSH values. Our data indicate that SHBG exhibited a modest increase within the reference range at TSH levels below the reference range. LDL-C was less dynamic and showed no significant variation with TSH. At TSH values above the reference range, LDL-C revealed a numerical elevation. This is in accordance with previous studies showing increased LDL-C levels in hypothyroidism (28), which by themselves may promote the development of atherosclerosis and cause cardiovascular disease. Only a few patients with suppressed TSH levels, however, exhibited abnormal elevations of the safety parameters PINP and NT-proBNP outside the reference range, further highlighting possible discrepancies between low TSH levels and pathological effects at the tissue level.

When comparing biomarkers between the three different treatment regimens, patients on T3 monotherapy showed a higher level of SHBG compared with T4 monotherapy or T4/T3 combination therapy. Previously, we reported lower LDL-C levels among groups of patients on T3 monotherapy compared to T4 monotherapy and this difference was corroborated in this study. The patients on T4 monotherapy revealed a slightly higher mean TSH of 0.65, vs. TSH levels of 0.33 and 0.29 in the T3 monotherapy and T4/T3 combination groups, respectively. This difference in TSH levels does not explain the differences observed, however, as regression analysis revealed that only 2.3% of the difference in SHBG and 3.0% of the difference in LDL-C, could be explained by the difference in TSH between groups.

Moreover, the distributions of NT-proBNP and PINP was similar between T4 monotherapy, T3 monotherapy and T4/T3 combination therapy. Our data therefore do not support the notion that T3 monotherapy has a safety profile different from T4 monotherapy or T4/T3 combination therapy, provided normal TSH levels are maintained. In terms of efficacy, however, the increased SHBG and reduced LDL-C levels seen in patients on T3 monotherapy andT4/T3 combination therapy vs. patients on T4 monotherapy further suggests, that a subgroup of patients on T4 monotherapy did not achieve the full cellular effect of the hormone dose they were administered.

When looking at the three treatment modalities investigated in this study: T4 monotherapy, T3 monotherapy and T4/T3 combination therapy the same pattern emerges. Higher efficacy parameters in patients receiving additional T3, but unchanged safety parameters in the middle of the reference range. This is significant in relation to the ongoing debate around T3 monotherapy, where concerns in relation to increased cardiovascular and skeletal risk have been raised. Our data suggest that patients receiving T3 monotherapy do not exhibit evidence of increased risk of cardiac or skeletal adverse effects compared to other treatment modalities according to the laboratory biomarkers evaluated. Moreover, the data suggest that assessment of cellular action of thyroid hormones using biomarkers may be superior to just relying on assessment of serum TSH in the peripheral circulation. From a biological point of view, this makes sense; the cellular action of thyroid hormone depends on sufficient binding of T3 to the nuclear T3-receptor. The amount of T3 reaching the nuclear receptor may be further modulated by transmembrane transport processes or intracellularly by deiodinases and other intracellular modifications. Thus, increased extracellular T3 concentrations may not result in pathologically high intracellular T3 levels. The biomarker levels assessed in this study would suggest this to be the case.

In conclusion, our findings further support SHBG as a dynamic biomarker of intracellular thyroid hormone action, whereas LDL-C appeared less responsive to variations in thyroid hormone status. Patients receiving T3 monotherapy exhibited higher SHBG levels and lower LDL-C levels than those receiving other treatment regimens, without corresponding increases in biomarkers reflecting potential adverse effects on the heart or skeleton. This suggests that in a subgroup of patients T3 monotherapy will further enhance the clinical effects of thyroid hormone therapy, without increasing the risk of adverse effects. However, longer studies are needed to assess both efficacy and long-term safety of T3 on monotherapy. The data emerging from this study highlight the complex interplay between thyroid hormones and their systemic impacts, emphasizing the importance of identifying markers beyond traditional TSH measurements.

## Data Availability

The raw data supporting the conclusions of this article will be made available by the authors, without undue reservation.
